# Synergistic Effect of Combined Mirror Therapy on Upper Extremity in Patients With Stroke: A Systematic Review and Meta-Analysis

**DOI:** 10.3389/fneur.2020.00155

**Published:** 2020-04-02

**Authors:** Zhonghua Luo, Yuqing Zhou, He He, Shanshan Lin, Rui Zhu, Zhen Liu, Jiemei Liu, Xiaoli Liu, Shuping Chen, Jihua Zou, Qing Zeng

**Affiliations:** ^1^First Clinical Medical College, Southern Medical University, Guangzhou, China; ^2^Department of Rehabilitation Medicine, Zhujiang Hospital, Southern Medical University, Guangzhou, China; ^3^Department of Rehabilitation Medicine, The First Affiliated Hospital, Sun Yat-sen University, Guangzhou, China; ^4^Department of Rehabilitation Medicine, The First People's Hospital of Foshan, Foshan, Guangdong, China; ^5^Department of Rehabilitation Medicine, Shunde Hospital, Southern Medical University, Guangzhou, China; ^6^School of Rehabilitation Medicine, Southern Medical University, Guangzhou, China

**Keywords:** mirror therapy, combined therapy, upper limb, stroke, functional recovery

## Abstract

**Background:** There is an increasing trend for researchers to combine mirror therapy with another rehabilitation therapy when treating the upper extremity of patients with stroke.

**Objective:** To evaluate the synergistic effect of combined mirror therapy (MT) on the upper extremity in patients with stroke and to judge efficacies of four combined mirror therapy subgroups [EMGBF group: electromyographic biofeedback (EMGBF) + MT; MG group: mesh glove (MG) + MT; AT group: acupuncture (AT) + MT; ES group: EMG-triggered electrical stimulation (ES) + MT].

**Methods:** CNKI, Wan Fang, VIP, Web of Science, ScienceDirect, PubMed, OVID LWW, and Cochrane were used. We searched these databases for randomized controlled trials published from January 2013 to August 2019, which presented results of combining mirror therapy with other rehabilitation therapies. Quality assessments were performed using the Cochrane Handbook criteria in order to accurately review interventions. The primary outcomes were measured by the Fugl–Meyer Assessment—upper extremity (FMA-UE).

**Results:** Ten trials, with a total of 444 patients whose upper limb functions were damaged after stroke, were included in the meta-analysis. Compared with the control group, a remarkable effect of combined mirror therapy [all: weight mean difference in random effects model (WMD): 8.07, 95% confidence interval (CI) 5.87, 10.26] on functional recovery of the upper limb was detected. However, a high value of heterogeneity (χ2 = 20.09, df = 9; *I*^2^ = 55%) was found. The subgroup analysis (EMGBF group: WMD = 8.95, 95% CI 6.33, 11.58; ES group: WMD = 10.14, 95% CI: 5.67, 15.01) showed moderate improvement in functional recovery of the upper extremity in patients with stroke when mirror therapy was combined with conventional therapy. Furthermore, no difference in efficacy on upper extremity in patients with stroke was observed between the EMGBF group and the ES group.

**Conclusion:** Despite the heterogeneity, the results indicate that combining mirror therapy with another rehabilitation therapy on the upper extremity in patients with stroke is better than single rehabilitation therapy. However, more randomized controlled clinical trials and larger sample sizes are required for an in-depth meta-analysis.

## Introduction

Stroke is one of the primary causes of disability not only in middle-aged but also elderly people worldwide (1). Stroke survival is often accompanied by paralysis of the upper and lower limbs, which seriously affects the quality of life of patients (2, 3). Therefore, rehabilitation therapy after stroke is very important.

Several current interventions are used to improve upper limb function, including mirror therapy (MT) (4, 5), constraint-induced movement therapy (6, 7), acupuncture (8), electromyographic biofeedback (EMGBF) (9), afferent stimulation (10), and robot-assisted therapy (11). Recently, the promising therapy, MT, is popular with researchers due to it being simple, cheap, and maneuverable. Among them, MT refers to the application of a simple device, called a “mirror box,” which uses the principles of the same object image and distance reflected by the plane mirror to replace the normal limb image, which achieves the rehabilitation goal of eliminating abnormal sensation and restoring motor function (12). For example, Ramachandran et al. first discovered that the mirror box could provide a useful new tool to reconstruct the sensory circuitry of phantom limbs (13). Stevens and Zeng et al. further found that in hemiplegia, the function of the damaged limb significantly improved within 3 months in hemiplegia, indicating the potential of using mirror therapy as a cognitive strategy for upper extremity functional recovery (14, 15). Yavuzer and Rothgangel et al. reported that the improvement of upper extremity with mirror therapy was obvious than with conventional treatment program (16, 17). To further improve treatment effect, researchers combined mirror therapy with another rehabilitation therapy on upper extremity in patients with stroke and found preliminary evidence that combined mirror therapy is more effective than pure rehabilitation therapy (18, 19). Therefore, in recent studies, researchers are focusing more on mirror therapy with the combination of electromyographic biofeedback, mesh glove, acupuncture, or EMG-triggered electrical stimulation applied for the rehabilitation of the upper extremity.

EMG-BF has been established as a significant treatment for all kinds of peripheral nerve injuries (PNI) (20, 21). It improves motor function by promoting proprioceptive feedback caused by cortical recombination and muscle contraction through sensory stimulation (22). As early as 1982, Basmajian et al. found that the myoelectric biofeedback treatment for stroke patients with hemiplegia can significantly improve the recovery of upper limb motor function in stroke patients with hemiplegia (23, 24). Mesh glove (MG), a type of whole-hand electrical afferent stimulation, has been demonstrated to reduce muscle hypertonia and modify voluntary motor control as well as increase wrist extension motion. Therefore, it is expected to improve the daily life ability of stroke patients with a chronic neurological deficit (25, 26). Studies have shown that MG is likely to play an important role in plastic changes in the primary motor cortex and have a long-term influence on motor cortical excitability (26, 27). Acupuncture (AT) plays an irreplaceable role in traditional Chinese medicine and has a history of more than 3,000 years of use in China (28). As a unique Chinese medicine treatment, it is widely used to improve movement, sensation, speech, and other neurological functions in stroke patients (29, 30). EMG-triggered electrical stimulation (EMG-ES) is a process to increase electrical stimulation, starting with stimulation of a specific motor and reaching a threshold for muscular contraction. In the EMG method, when activity reaches the threshold for muscular contraction, the patient receives an additional electrical stimulus until there is maximum extension of the wrist several times to determine the target stimulation (18, 31). These four treatments have respective advantages and complement each other. Thus, the mirror therapy combination is regarded as a promising strategy for the treatment of the upper extremity in patients with stroke.

However, data is still not completely accurate, and further studies are still necessary. The aim of this meta-analysis is to investigate the synergistic effect of mirror therapy combined with other rehabilitation therapies on the upper extremity in patients with stroke, to screen for more effective rehabilitation methods for patients.

## Methods

### Data Sources and Search Strategy

According to the guidelines for randomized controlled trials provided by the Cochrane systematic evaluation of interventions, we systematically searched for studies published from January 2013 to August 2019 in the following databases: CNKI, VIP, Wan Fang, Web of Science, ScienceDirect, PubMed, OVID LWW, and Cochrane library.

### Quality Appraisal

To ensure the reliability of the included studies, two independent authors screened each study to assess quality using the criteria of the Cochrane Handbook (update 15.1.0) and the PEDro scale for reviewing interventions. The risk assessment criteria in the Cochrane Handbook are as follows (32): random sequence generation (selection bias), allocation concealment (selection bias), blinding of participants and personnel (performance bias), incomplete outcome data (attribution bias), selective outcome reporting (reporting bias), and other source of bias. The PEDro scale contains 11 items: inclusion criteria, random allocation, allocation concealment, baseline similarity, blinded subjects, therapist and referees, recording the key findings of 85% of the subjects, completing the target therapy, intergroup analysis, and primary outcome. Before the two authors evaluated the quality of studies, they studied the manuals, discussed differences in their views, and reached a consensus. When the two authors finished quality appraisal, a third professor made the final evaluation.

### Inclusion and Exclusion Criteria

#### Types of Studies

Randomized controlled clinical trials (RCTs) that combined mirror therapy with another rehabilitation therapy on the upper extremity in patients with stroke were examined.

#### Types of Participants

The enrolled patients were not restricted by age, gender, or area of limb hemiplegia (Tables 1, 2). Patients were eligible for inclusion if they (i) suffered from stroke in subacute or chronic phases according to diagnostic guidelines updated by the American Heart Association/American Stroke Association (38); (ii) had ≤to 46 points according to the Fugl–Meyer Assessment—upper extremity (FMA-UE) (39, 40); (iii) were able to comprehend and execute the therapeutic schedules; and (iv) were diagnosed with ischemic or hemorrhagic stroke for the first time. They were excluded if they (i) were diagnosed with severe cognitive impairment; (ii) suffered from other severe diseases such as brain tumor or brain trauma; or (iii) were also involved in other trials.

**Table 1:** Detailed description of 10 studies.

**References**	**ALL_n**	**Age, years, mean (SD)(E/C)**	**Paretic side (n: right/left)(E/C)**	**Time since stroke onset, day, mean (SD)(E/C)**	**Clinical stage**	**Severity (Brunnstrom stages) (E/C)**	**Type**	**Interventions(E/C)**
Wang and Chen (33)	60	47.02 ± 9.1/48.08 ± 10.2	11/19 10/20	119.26 ± 41.08/120.37 ± 39.4	Subcute	Unclear	EMGBF	EMGBF+MT+CT/EMGBT+CT
Xu (34)	40	62.40 ± 8.61/60.65 ± 8.80	13/7 12/8	17.61 ± 7.63/15.36 ± 8.19	Subcute	1.15 ± 0.37/1.20 ± 0.41	EMGBF	EMGBF+MT+CT/EMGBF+CT
Yao (21)	60	57.40 ± 7.323/57.07 ± 6.181	14/16 14/16	18.03 ± 5.654/21.23 ± 8.365	Subcute	2.93 ± 1.0065/2.67 ± 1.011	EMGBF	EMGBF+MT+CT/EMGBF+CT
Xie et al. (35)	90	56 ± 8/54 ± 6	27/18 21/24	40.73 ± 6.75/42.69 ± 7.42	Subcute	Unclear	AT	AT+MT+CT /MT+CT
Zhang et al. (36)	40	55.2 ± 10.9/54.9 ± 11.3	11/9 8/12	19.6 ± 20.3/30.8 ± 28.7	Subcute	2.15 ± 0.726/1.9 ± 0.70	AT	AT+MT+CT /MT+CT
Zhou and Ye (30)	40	57.22 ± 6.15/54.20 ± 5.03	unclear	<8 weeks	Subcute	Unclear	AT	AT+MT+CT /MT+CT
Lin et al. (37)	28	55.79 ± 14.59 /56.01+12.53	8/6 6/8	158.97 ± 95.34/129.5 ± 81.27	Chronic	4.25 ± 0.64 /4.25 ± 0.64	MG	MG+MT+CT/MT+CT
Lee et al. (10)	32	52.50+13.24 /56.64+9.43	7/8 7/10	660 ± 421.2 /531.3 ± 397.2	Chronic	Unclear	MG	MG+MT+CT /MT+CT
Kim et al. (31)	23	55.92 ± 11.75/55.64 ± 12.61	4/8 6/5	34.06 ± 1.65/35.00 ± 15.05	Subcute	3.5 ± 0.97/3.28 ± 1.051	ES	ES+MT +CT /ES+CT
Schick et al. (18)	32	62 ± 19.6 /63 ± 11.5	7/8 8/9	1–6 months	Subcute	Unclear	ES	ES+MT+CT/ES+CT

**Table 2:** Detailed description of 10 studies (continued Table 1).

**Duration**	**Case_n**	**Case_mean**	**Case_SD**	**Control_n**	**Control_mean**	**Control_SD**	**Duration(min)**	**Outcome measures**
5 × 30 min sessions over a 4-week period	30	38.97	10.06	30	33.17	10.49	600	FMA;AROM;IEMG;
6 × 40 min sessions over a 8-week period	20	34.3	6.31	20	23.8	5.09	1920	BN;FMA;MAS;
6 × 20 min sessions over a 4-week period	30	51.2	7.871	30	42.23	11.316	480	BN;FMA;FIM;
MT:5 × 30 min sessions over a 4-week period AT:5 × 30 min sessions over a 4-week period	45	45.96	4.03	45	38.58	1.98	900	FMA;BI;STEF;
AT:6 × 20 min sessions over a 4-week period AT+MT: 6 × 20 min sessions over a 4-weekperiod	20	47.7	9.71	20	32.7	8.73	480	FMA;AROM;BI;BN;
5 × 30 min sessions over a 12-week period	20	34.97	7.85	20	25.71	9.45	1800	FMA;BI;
5 × 90min sessions over a 4-week period	14	50.93	9.41	14	49.86	8.97	1800	FMA;Myoton;BBT;10 MWT;MAL;
5 × 90 min sessions over a 4-week period	15	43.6	9.76	16	43.56	8.73	1800	FMA; FIM; rNSA; BBT;
5 × 40 min sessions over a 3-week period	12	26.67	8.68	11	17.45	5.69	600	BBT;FMA;BN;MFT;
5 × 30 min sessions over a 3-week period	15	29.73	14.4	17	17.73	9.1	450	FMA;

*E, experimental group; C, control group; EMGBF, Electromyographic biofeedback; AT, Acupuncture; MG, Mesh glove; ES, EMG-triggered electrical stimulation; CT, Conventional therapy; MT, mirror therapy; FMA, Fugl-Meyer Assessment; AROM, active range of motion; IEMG, Imaging electromyography; BN, Brunnstrom stage; MAS, motor assessment scale; FIM, Function Independence Measure; MBI, Modified Barthel Index; STEF, simple test for evaluating hand function; BI, Barthel Index; BBT, Box and Block Test; 10MWT, 10-Meter Walk Test; MAL, MAL, Motor Activity Log.; rNSA, revised Nottingham Sensory Assessment; MFT, Manual Function Test; n, number; mean, average number; SD, standard difference*.

#### Types of Intervention

Combined mirror therapy was compared with single rehabilitation therapy, and all the patients received conventional therapy. There are four combined therapies such as EMGBF + MT, AT + MT, ES + MT, and MG + MT. Since the experimental scheme of each combined method is different, the strategies of classifying it into a class of the same methods are (i) the same principle of experiment; (ii) target group consistency; (iii) using an identical single-blind method; (iv) had initiative in moving their impaired upper extremity or moved assisted by therapist in order to be in line with unaffected extremity.

### Outcome Measures

FMA-UE, as a professional assessment, was used to measure the outcome in the upper limb's functional recovery in terms of reflex ability, synergic movement, wrist stability, and hand grip strength.

### Search Strategies

All the searches were performed in electronic databases published in English or Chinese, specifically CNKI (publication year: 2013.01.01–2019.08.01; language: Chinese and English; all types of literature), PubMed (publication date: 2013.01.01–2019.08.01; language: English; all types of literature); Wan Fang (date of publication: 2013–2019; article types: paper), Web of Science (time span: 2013–2019), ScienceDirect (years: 2013–2019; all types of articles), SpringerLink (show documents published: between 2013 and 2019), OVID LWW (publication year: 2013.2018), Cochrane library (trials; publication year: between 2013 and 2018). There were three key words used to search the literature, namely (“upper limb” or “upper extremity” or “membrum superius” or “pectoral limb”) AND (“stroke” or “cerebrovascular stroke” or “cerebrovascular accident”) AND (“mirror therapy”).

### Data Collection and Exclusion

The results of the literature search were brought into the CNKI E-study, and duplicate records were removed. One author reviewed and assessed the title, abstract, and purpose of the document to remove irrelevant studies. After this preliminary screening, two independent authors filtered the remaining results according to (i) clear outcome; (ii) combined therapy; (iii) completed data; (iv) outcome assessment of FMA-UE; (v) randomized controlled trial; and (vi) single blind or double blind. After discussion and negotiation, 10 studies were included in the quantitative synthesis (meta-analysis).

### Data Extraction

Blinded to the journal, we made a detailed form (Tables 1, 2) based on PRISMA that described the enrolled studies' characteristics in terms of publication year, sample size, author, and patient characteristics [i.e., age, paretic side, severity (Brunnstrom stages), time when patient was diagnosed with a stroke, interventions (i.e., intervention types and duration), outcome measures and statistic data (i.e., case group's number (n); case group's mean; case group's standard difference (SD); control group's n; control group's mean; control group's SD)]. When we encounter problems, we contacted the first author by email as much as possible.

### Data Analysis

To accurately infer the synergistic effect of combined mirror therapy for functional recovery in a stroke patient's upper limb, raw data from research materials were processed using Review Manager 5.3 and Stata 12.0 to calculate weight mean difference (WMD) with a confidence interval of 95% (95% CI). Given the continuity of the data, the best methods random effects model and the statistical method of inverse variance were, respectively, used to compare combined therapy with single rehabilitation therapy. The weight mean difference (WMD) and 95% confidence intervals (95% CI) were used to assess the mean effect size of therapy. Heterogeneity among studies was assessed using *I*^2^ tests (a value of *p* < 0.1 was considered to indicate the existence of significant heterogeneity) and chi-square (0–40% low; 40–60% moderate; 60–100% high heterogeneity). Subgroup analysis (1) combined therapy subgroup: EMGBF group, ES group, AT group, and MG group, and (2) the subgroup's control method: (i) adding mirror therapy to rehabilitation therapy in the experimental group. (ii) adding rehabilitation therapy to mirror therapy in the experimental group) was performed using Review Manager 5.3. In order to investigate the sources of heterogeneity, we rigorously applied moderator analyses using Stata12.0 (i.e., meta-regression and publication bias) (41). Differences were considered statistically significant when the *p* < 0.05.

## Results

**Figure 1:**
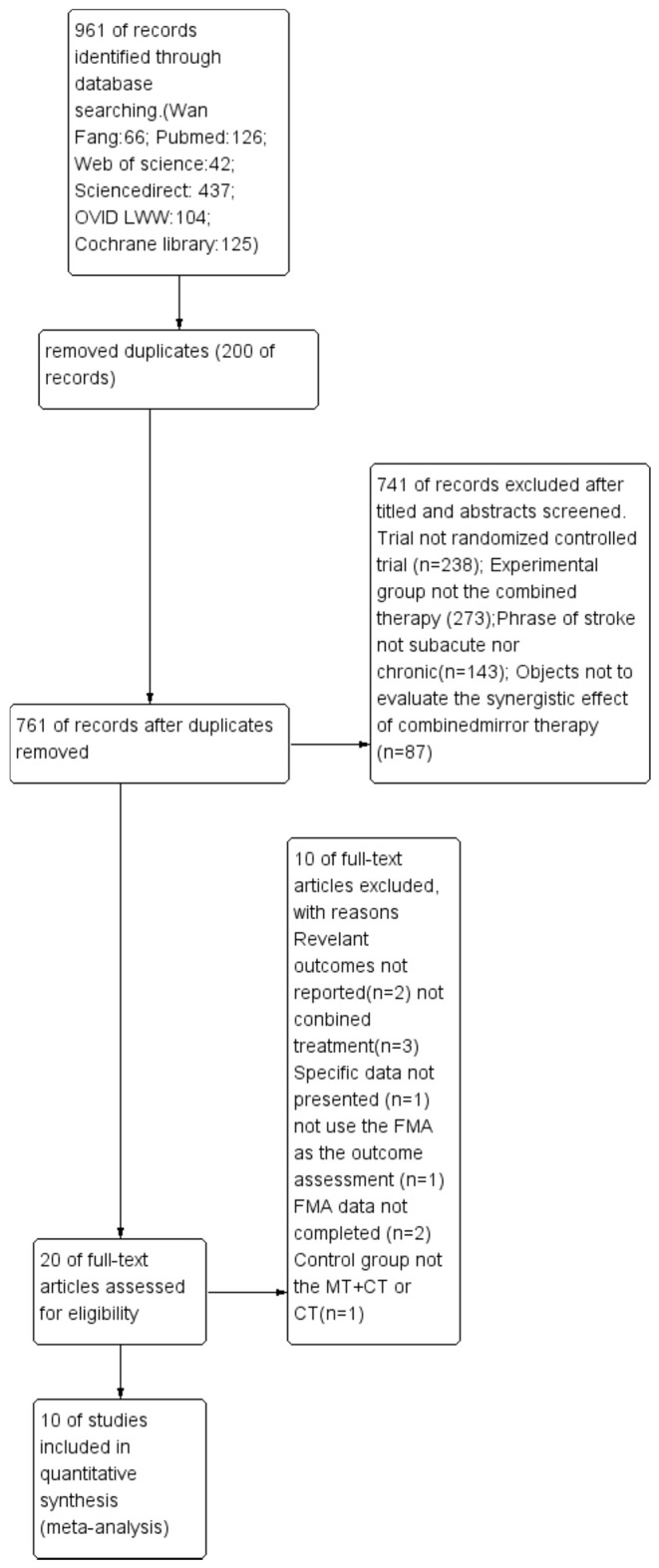
Literature search and study selection. FMA: Fugl-Meyer Assessment; MT: mirror therapy; CT: combined therapy.

**Figure 2:**
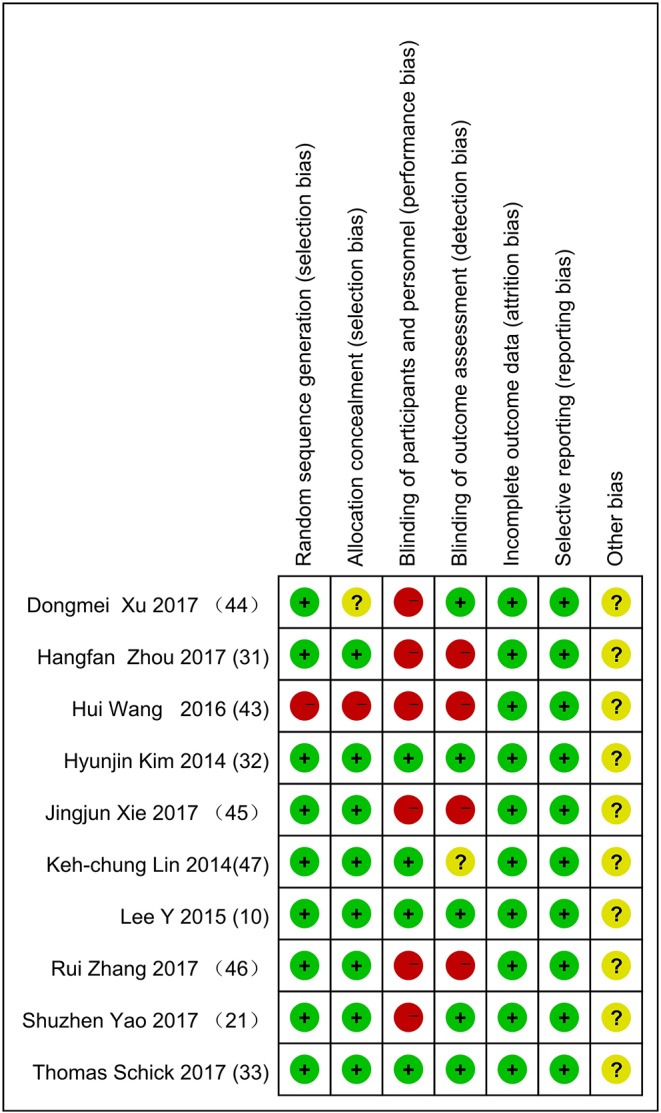
Authors' judgments about each risk of bias item for included studies.

Nine hundred sixty-one records were identified through database searching, and 761 records were retained after removal of duplicates. In the end, 10 studies (10, 21, 31–33, 43–47) were included in the quantitative synthesis (meta-analysis). The detailed process for selecting studies is demonstrated in Figure 1. Studies published between 2013 and 2019 were included in the meta-analysis. A total of 444 patients were studied, with 221 patients in the experimental group and 223 patients in the control group. Tables 1, 2 summarize the 10 studies in detail. The average age of the patients ranged from 47.02 to 63.00 years. The mean time since stroke onset was 15.36 to 6 months except for two studies (10, 47) whose onset time of stroke was more than 6 months. Five studies (21, 32, 44, 46, and 47) precisely described the average Brunnstrom stages, which ranged from 1.15 to 4.25. The duration of interventions was from 450 to 1,920 min. Figure 2 presents the authors' judgments about the risks of bias for the included studies. All studies (10, 21, 31–33, 43–47) described the methods used to generate the allocation sequence in sufficient detail, and all studies had complete data. The risk of selection bias (allocation concealing) was obscure in five studies (21, 31, 32, 43, and 46) because of insufficient information, and the selection bias (allocation concealing) of Xu (44) was considered high due to the allocation sequence being generated by date of admission. Performance bias (blinding of participants and personnel) was low in six studies (10, 21, 33, 44, 46, and 47) because reliable blinding methods were implemented for both participants and study personnel, while these factors were obscure in four studies (31, 32, 43, and 45). Detection biases (detection of outcome assessment) were not able to be estimated for three studies (32, 43, and 45) as no information was given. Table 3 shows the gross score for each study in the internal validity analysis carried out using the PEDro scale: four studies were excellent (>8), five studies were good (≥6, ≤8), and one study was fair (≥4, ≤5).

**Table 3:** Internal validity analysis.

**References**	**2**	**3**	**4**	**5**	**6**	**7**	**8**	**9**	**10**	**11**	**Total**
Wang and Chen (33 )	-	-	•	-	-	-	•	•	•	•	5
Xu (34)	•	•	•	-	-	-	•	•	•	•	7
Yao (21)	•	•	•	-	-	•	•	•	•	•	8
Xie et al. (35)	•	•	•	-	-	-	•	•	•	•	7
Zhang et al. (36)	•	•	•	-	-	-	•	•	•	•	7
Zhou and Ye (30)	•	•	•	-	-	-	•	•	•	•	7
Lin et al. (37)	•	•	•	•	-	•	•	•	•	•	9
Lee et al. (10)	•	•	•	•	-	•	•	•	•	•	9
Kim et al. (31)	•	•	•	•	•	•	•	•	•	•	10
Schick et al. (18)	•	•	•	•		•	•	•	•	•	9

**Figure 3:**
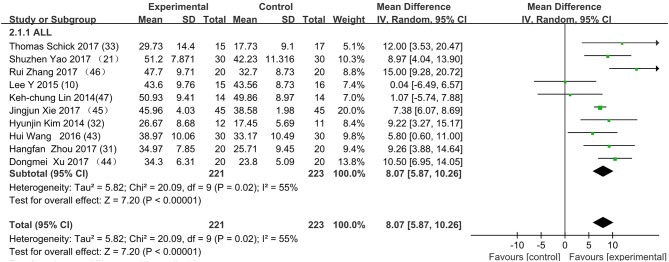
Forest plot of the random effects meta-analysis of CT and MT on motor function of the upper extremity. SD: standard deviation; 95% CI: 95% confidence interval.

**Figure 4:**
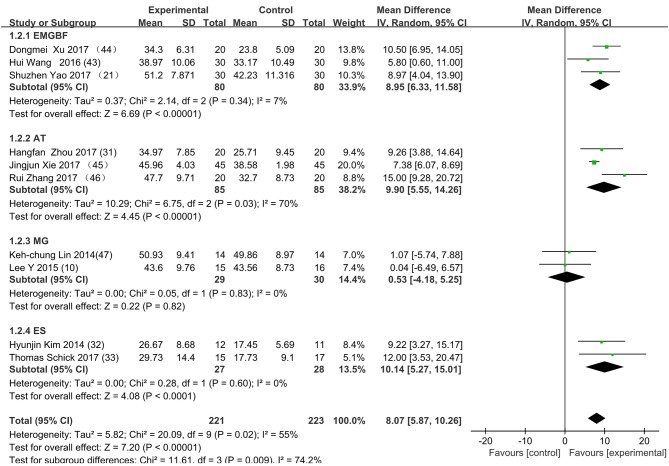
Subgroup analysis for the high heterogeneity. SD: standard deviation; 95% CI: 95% confidence interval. EMGBF: Electromyographic biofeedback; AT: Acupuncture; MG: mesh glove; ES: electrical stimulation.

**Figure 5:**
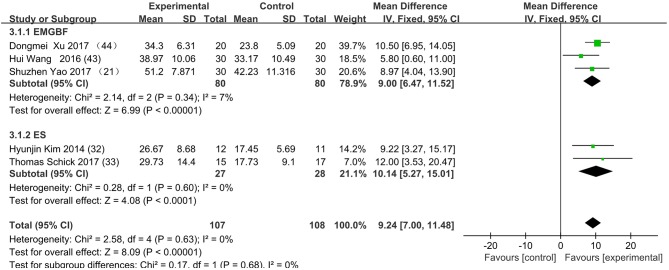
The subgroup's control method is adding MT to rehabilitation therapy in experimental group. EMGBF group: (E: EMGBF+MT/C: EMGBT); ES group: (E: ES+MT/C: ES) E: experimental group; C: control group.

**Figure 6:**
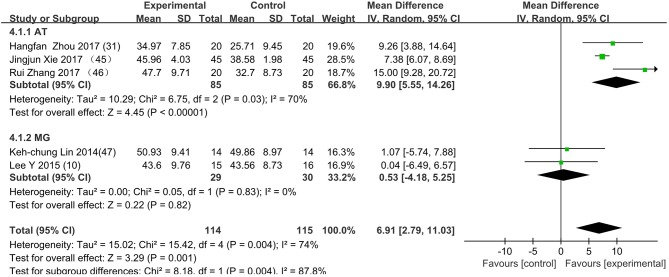
The subgroup's control method is adding rehabilitation therapy to MT in experimental group. AT group: (E: AT+MT/C: MT); MG group: (E: MG+MT/C: MT); E: experimental group; C: control group.

**Figure 7:**
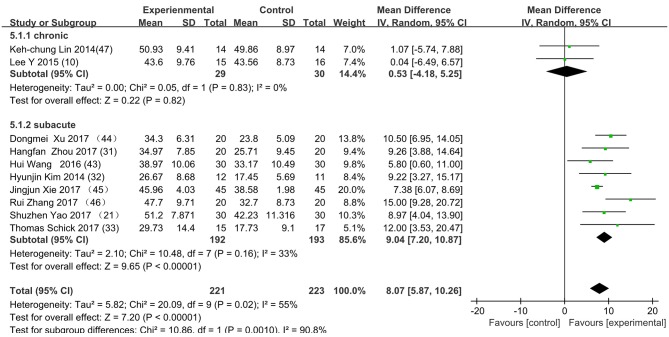
Subgroup analysis for discriminating between subacute or chronic stroke.

Figure 3 presents the random effects meta-analysis of mirror therapy (MT) combined with another rehabilitation therapy and applied to functional recovery of a stroke patient's upper limb. Using the standard chi square test, the heterogeneity statistic (χ^2^ = 20.09, *p* = 0.02; *I*^2^ = 55%) was significant. The value for overall effect is 7.20 (*p* < 0.00001) in random mode due to the existence of substantial heterogeneity, and the total weight mean difference were 8.07 (95% CI: 8.07, 10.26). Meanwhile, a subgroup analysis (Figure 4) was applied to detect the cause of high heterogeneity, and this revealed that the AT group (*I*^2^ = 70%) was the important factors. Figure 4 shows that the EMGBF group (WMD = 8.95, 95% CI: 6.33, 11.58) and ES group (WMD = 10.14 95% CI: 5.67, 15.01) showed moderate improvement in functional recovery on upper extremity in patients with stroke, but no difference was witnessed in the MG group (WMD = 0.53, 95% CI −4.18, 5.25, Z = 0.22, *p* = 0.82). The difference between the subgroup analysis in Figures 5, 6 is the interventional method adding mirror therapy to rehabilitation therapy in the experimental group (Figure 5) or adding rehabilitation therapy to mirror therapy in the experimental group (Figure 6). No difference in efficacy on upper extremity in patients with stroke was observed between the EMGBF group and ES group in Figure 5. Figure 6 shows that there is a substantial heterogeneity (χ^2^ = 15.42, *I*^2^ = 74%) and a subgroup difference (χ^2^ = 8.18, *I*^2^ = 87.8%) between the AT group and MG group. Meta-analysis regression (Table 4) was used to examine the cause of high heterogeneity, with inconclusive results: the covariate sample size (*p* > 0.352) and during treatment (*p* > 0.782) showed significant correlation with high heterogeneity. Finally, an Egger test (coefficient = 0.2267264; 95% CI: −1.687296, 2.140749; *p* = 0.792) showed no sign of publication bias among the 10 studies (Table 5). The subgroup analysis (Figure 7) was applied to analyze the relationship between the time elapsed since stroke onset and the high heterogeneity. Figure 7 shows that there is a substantial subgroup difference (χ^2^ = 10.86, *I*^2^ = 90.8%) between the chronic group and subacute group.

**Table 4:** Results of meta-analysis regression.

**Covariance**	**Coefficients**	**Standard error**	***t***	**P>|t|**	**95% CI**
ALL_n	0.0294893	0.292273	1.01	0.352	(−0.0420273,0.1010059)
duration	−0.0004548	0.0015713	0.29	0.782	(−0.0033899,0.0042995)
_cons:	0.7778708	3.402441	−0.23	0.827	(−9.103343,7.547602)

**Table 5:** Results of publication bias.

**Std_Eff**	**Coefficients**	**Standard error**	***t***	**P>|t|**	**95% CI**
Slope	7.474338	1.419139	5.27	0.001	(4.201797,10.74689)
Bias	0.2267264	0.830017	0.27	0.792	(−11.46154,2.140749)

## Discussion

This is the first meta-analysis probing the synergistic effect of combined mirror therapy on the upper extremity in patients with stroke. Some preliminary conclusions can be drawn from this meta-analysis. First and foremost, this meta-analysis of 10 RCTs including 444 patients showed that combined mirror therapy (mirror therapy mixed with other rehabilitation therapies) was superior to single rehabilitation therapy to promote upper limb motor function of stroke patients (WMD 8.07, 95% CI 5.87, 10.26) in terms of muscle reflex ability, coordinated movement, and accurate operation in the Fugl–Meyer Assessment (FMA). However, heterogeneity (χ^2^ = 20.09, *p* < 0.00001; *I*^2^ = 55%) was high, and one study [Lee (10)] did not draw a precise conclusion about whether combined mirror therapy (mirror therapy with MG therapy) was better than pure mirror therapy in promoting upper limb motor function. The difference between Lee's study and the other studies is that the stimulation intensity—other studies (37) were at the sensory threshold of the non-operatic hand (20 Hz, with pulse rate of 300 μs), but the MG intensity in this study was set at the sensory threshold of the paretic hand (50 Hz, with a pulse rate of 300 μs). Further evidence is needed to determine whether sensory threshold leads to significant differences. From the subgroup analysis (*I*^2^: EMGBF group 7%; AT group 70; MG group 0%; ES0%), it is clear that the high heterogeneity came from the AT group as expected. A further subgroup analysis separated interventional methods: adding mirror therapy to rehabilitation therapy in the experimental group (Figure 5) and adding rehabilitation therapy to mirror therapy in the experimental group (Figure 6). Figure 5 showed that the synergistic effect of combining mirror therapy with EMGBF was the same as that of combining mirror therapy with ES. In Figure 6, it is difficult to judge whether AT + MT has an advantage over single treatment due to the high heterogeneity. The time since stroke onset is likely to cause the high heterogeneity because the mean time in the Xie study (40.73 ± 6.75/42.69 ± 7.42 days) was longer than in the Zhang study (19.6 ± 20.3/30.8 ± 28.7 days). It is likely that the more early patients received AT + MT, the upper limb function will be more effectively improved. A large sample size is necessary to verify this hypothesis. Meanwhile, MG + MT, a popular treatment abroad, showed no significant effect in promoting upper limb motor function in stroke patients in this meta-analysis. This finding is inconsistent with those of Peurala et al. (25) and Dimitrijevic, wherein MT combined with MG stimulation provided additional benefits for manual dexterity when compared with MT alone. Because a string of studies had demonstrated that MG could effectively improve upper limb motor function in stroke patients, meta-analysis regression was applied to detect the reason for this discrepancy. However, neither sample size (*p* > 0.186) nor duration of treatment (*p* > 0.787) could be regarded as the cause of high heterogeneity. The result was discussed in correspondence with Wen Zeng (15) whose meta-analysis mainly explored mirror therapy on motor function of the upper extremity in patients with stroke. From this discussion, the conclusion that two factors (sample size and duration of treatment) were regarded as the cause of high heterogeneity can be reached. Figure 7 shows the significant effect of sample size and duration of treatment on the subacute group compared with that on the chronic group, and the high heterogeneity found in the subgroup analysis was related to the time elapsed since stroke onset.

There were many factors not detected in the studies included in this meta-analysis, such as paretic side, severity (Brunnstrom stages), age, and sex, resulting in incomplete data in Table 1. For instance, the details of the paretic side were not described in Hangfan Zhou (31). No evidence in recent years has demonstrated a relationship between paretic side and treatment, and this unknown area should be explored by researchers. Table 1 also shows that Wang (43), Xie (45), Zhou (31), Lee (10), and Schick (33) did not describe the details of severity (Brunnstrom stages), which limited the quality of the articles. Safaz (42) and Watanabe (43) had confirmed that BRS (Brunnstrom stages) is a convenient and effective tool for the evaluation of UEs in early stage stroke patients. Besides these factors, there may be unknown elements contributing to the high level of heterogeneity in publication bias. Wang (43) and Xu (44) did not describe the details of allocation group concealing, which can lead to selection bias. In addition, Yao (21), Zhou (31), Wang (43), Xie (45), and Zhang (46) did not describe the details of blinding of participants and personnel, and implementation bias can arise when participants and implementers are aware of the interventions. Further, Zhou (31), Wang (43), Xie (45), Zhang (46), and Lin (47) did not describe the details of blinding of intervention allocation in outcome assessment, which can lead to measurement bias.

There are several limitations of this study that should be taken into account. First, the number of studies included in meta-analysis was limited, reducing the representativeness of the article. This was unavoidable due to the particularity of topic selection, the limitation of resources, and the rigor of the article. Second, the high heterogeneity of the studies partly limits the impact of this paper. The objective of this meta-analysis is to study combined therapy, focusing on mirror therapy mixed with other therapies such as AT, ES, EMBGF, and MG, so the high heterogeneity is unavoidable. Third, studies published in English and Chinese were included in the analysis, but studies in other languages were not included. Fourth, all articles were randomized controlled trials, but there is a belief that non-randomized controlled trials should also be taken into account when RCTs are unfeasible or unethical.

From a patient's perspective, we must take expense and time spent on combined mirror therapy into consideration. If there is a directly proportional relationship between expense and efficacy on recovery, we might as well take combined therapy as first choice for patients after stroke. In summary, combining mirror therapy with another rehabilitation therapy (especially electromyographic biofeedback and EMG-triggered electrical stimulation) is better than single rehabilitation therapy on upper extremity in patients with stroke. In the future, there should be considerable work applied by researchers to more deeply probe the optimal specific combination therapy.

## Data Availability Statement

All datasets generated for this study are included in the article/supplementary material.

## Author Contributions

JZ and QZ: conception and design, drafting the article. HH, SL, and RZ: acquisition of data. ZL and YZ: analysis and interpretation of data, editing the article. JL, XL, and SC: study supervision and revising the article. ZL and YZ contributed equally to this work and should be considered co-first authors. All authors proofed and approved the submitted version of the article.

### Conflict of Interest

The authors declare that the research was conducted in the absence of any commercial or financial relationships that could be construed as a potential conflict of interest.
